# Supplementary material for: The SWine IMputation (SWIM) haplotype reference panel enables nucleotide resolution genetic mapping in pigs
Source: Commun Biol. 2023 May 30;6:577. doi: 10.1038/s42003-023-04933-9 (PMC10229620; doi:10.1038/s42003-023-04933-9)
Supplement: Supplementary file 2 — Supplementary Information [file 42003_2023_4933_MOESM2_ESM.pdf]

Supplementary Information

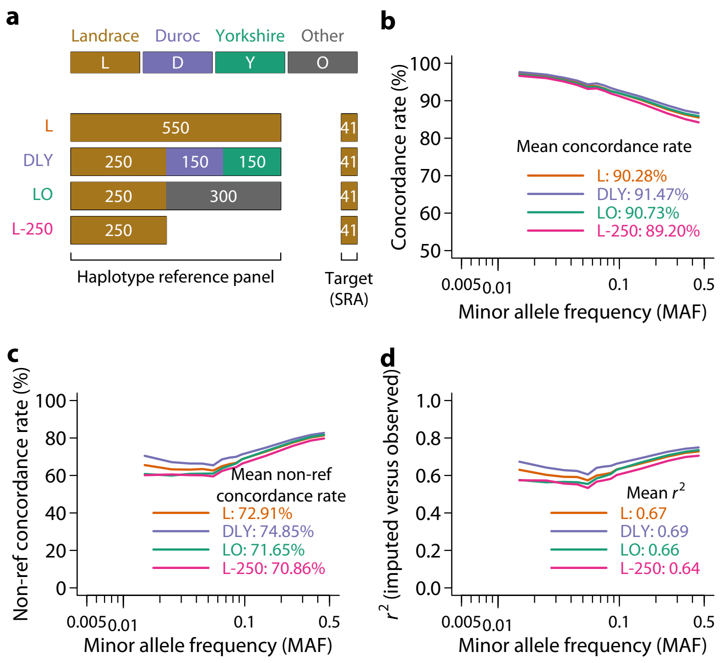

Supplementary Figure 1 | Effects of breed composition of haplotype reference panel on imputation accuracy. (**a**) Experimental design to investigate the effect of breed composition of haplotype reference panel on imputation accuracy. Three reference panels were tested, including 'L': 550 Landrace animals; 'DLY': 550 pigs from the Duroc, Landrace, and Yorkshire breeds; 'LO': 550 pigs from Landraces and other non-Duroc or Yorkshire breeds; 'L-250': 250 Landrace animals only. 41 Landraces from the SRA were used as the target set. Concordance rate (**b**), non-reference concordance rate (**c**) and r^2 (**d**) of imputed versus observed genotypes using different breed composition of the haplotype reference panel.

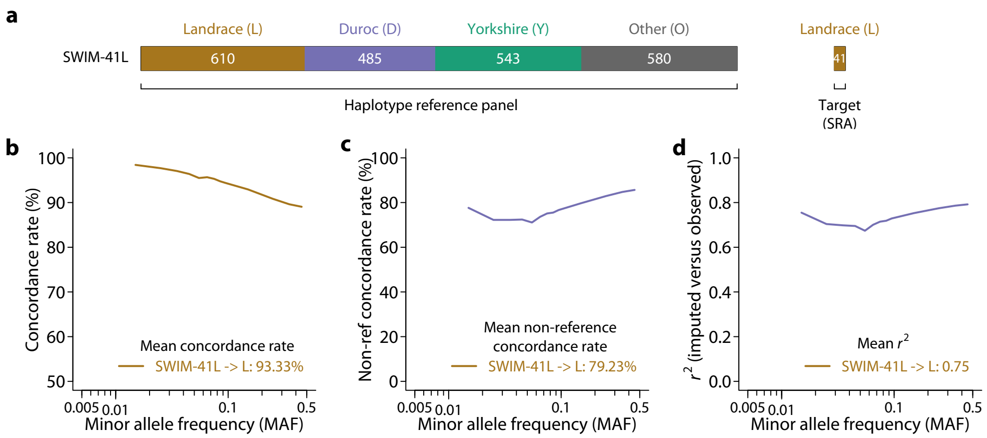

Supplementary Figure 2 | Imputation accuracy using a large multi-breed panel on genetically distant individuals. (**a**) Experimental design where the entire SWIM reference panel minus the 41 Landraces were used as the reference and the 41 Landrace animals from the SRA as target. Concordance rate (**b**), non-reference concordance rate (**c**) and r^2 (**d**) of imputed versus observed genotypes.

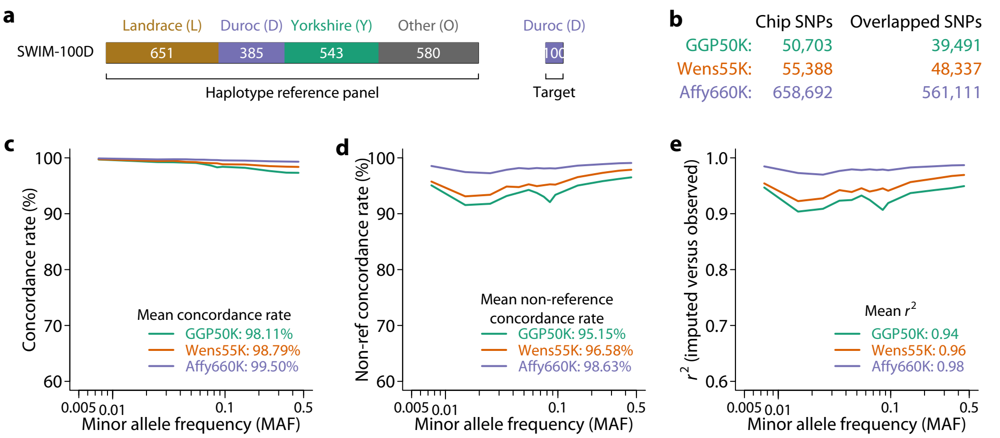

Supplementary Figure 3 | Effects of starting SNP chips on imputation accuracy. (**a**) Experimental design where the entire SWIM reference panel minus the 100 Durocs were used as the reference and the 100 Durocs as target. (**b**) Number of SNPs on each chip and overlapped with the SWIM reference panel. The numbers are for autosomal SNPs only. Concordance rate (**c**), non-reference concordance rate (**d**) and r^2 (**e**) of imputed versus observed genotypes using different breed composition of the haplotype reference panel.

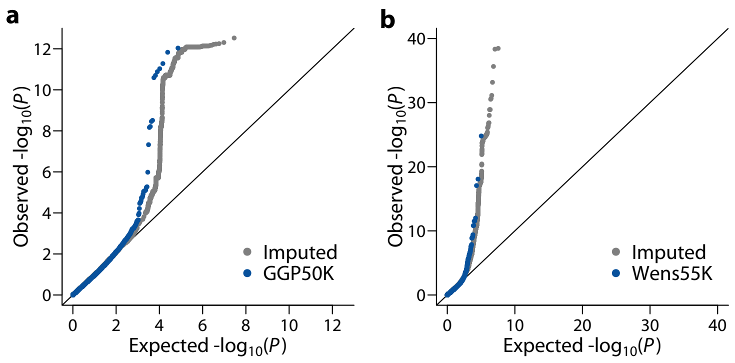

Supplementary Figure 4 | QQ plots of GWAS p values QQ plots of GWAS P values for backfat thickness (**a**) and body length (**b**).
