# Supplementary material for: The SWine IMputation (SWIM) haplotype reference panel enables nucleotide resolution genetic mapping in pigs
Source: Commun Biol. 2023 May 30;6:577. doi: 10.1038/s42003-023-04933-9 (PMC10229620; doi:10.1038/s42003-023-04933-9)
Supplement: Supplementary file 3 — Description of Additional Supplementary Files [file 42003_2023_4933_MOESM3_ESM.pdf]

Description of Additional Supplementary Data

File Name: Supplementary Data 1

Description: Sources of sequence data used in this study for analysis and imputation.

File Name: Supplementary Data 2

Description: Accession numbers for SRA samples used in this study.

File Name: Supplementary Data 3

Description: GWAS signficant SNPs ($P < 5 \times 10^{-8}$) for backfat thickness using SNP chip.

File Name: Supplementary Data 4

Description: GWAS signficant SNPs ($P < 5 \times 10^{-8}$) for backfat thickness using imputed genotypes.

File Name: Supplementary Data 5

Description: GWAS signficant SNPs ($P < 5 \times 10^{-8}$) for body length using SNP chip.

File Name: Supplementary Data 6

Description: GWAS signficant SNPs (P < 5 x 10-8) for body length using imputed genotypes.

File Name: Supplementary Data 7

Description: Source data for Figure 1a.

File Name: Supplementary Data 8

Description: Source data for Figure 1b.

File Name: Supplementary Data 9

Description: Source data for Figure 2.

File Name: Supplementary Data 10

Description: Source data for Figure 3.

File Name: Supplementary Data 11

Description: Source data for Figure 4.

File Name: Supplementary Data 12

Description: Source data for Figure 6c.
